# Analyzing Adherence to Prenatal Supplement: Does Pill Count Measure Up?

**DOI:** 10.1155/2010/631971

**Published:** 2010-02-04

**Authors:** Kristie E. Appelgren, Paul J. Nietert, Thomas C. Hulsey, Bruce W. Hollis, Carol L. Wagner

**Affiliations:** ^1^Department of Pediatrics, Duke University, Durham, NC 27710, USA; ^2^Department of Biostatistics, Bioinformatics, and Epidemiology, Medical University of South Carolina, Charleston, SC 29425, USA; ^3^Division of Neonatology, Department of Pediatrics, Medical University of South Carolina, Charleston, SC 29425, USA

## Abstract

*Objective*. To determine if adherence as measured by pill count would show a significant association with serum-based measures of adherence. *Methods*. Data were obtained from a prenatal vitamin D supplementation trial where subjects were stratified by race and randomized into three dosing groups: 400 (control), 2000, or 4000 IU vitamin D_3_/day. One measurement of adherence was obtained via pill counts remaining compared to a novel definition for adherence using serum 25-hydroxy-vitamin D (25-OH-D) levels (absolute change in 25(OH)D over the study period and the subject's steady-state variation in their 25(OH)D levels). A multivariate logistic regression model examined whether mean percent adherence by pill count was significantly associated with the adherence measure by serum metabolite levels. *Results*. Subjects' mean percentage of adherence by pill count was not a significant predictor of adherence by serum metabolite levels. This finding was robust across a series of sensitivity analyses. *Conclusions*. Based on our novel definition of adherence, pill count was not a reliable predictor of adherence to protocol, and calls into question how adherence is measured in clinical research. Our findings have implications regarding the determination of efficacy of medications under study and offer an alternative approach to measuring adherence of long half-life supplements/medications.

## 1. Introduction

Vitamin D is an important nutrient that is widely known to be vital to bone health and development, although it has recently been linked to other systems such as immune function [1, 2]. The two main human sources of vitamin D are sunlight exposure, which converts 7-dehydrocholesterol in the skin to vitamin D_3_, and oral intake. Due to the limited dietary sources of vitamin D, serum vitamin D levels are primarily determined by sunlight exposure. The amount of vitamin D produced by a given amount of exposure is modified by skin pigmentation, with darkly-pigmented populations producing significantly less vitamin D than fair-skinned populations after exposure to similar conditions [3–7].

There is a strong relationship between maternal and fetal (cord blood) circulating 25(OH)D levels [8–11]. At the time of birth, cord blood as a direct reflection of fetal vitamin D status will contain approximately 50–60% of the maternal circulating levels of 25(OH)D. This relationship appears to be linear even at pharmacological intakes of vitamin D [12]. With respect to the more polar metabolites of vitamin D, a similar (but lesser) relationship is observed between mother and fetus [11]. Interestingly, there appears to be little, if any, relationship with respect to the parent vitamin, vitamin D [11]. This lack of placental transfer of the parent vitamin D from mother to fetus also has been observed in a porcine experimental animal model [13]. Thus, in the human fetus, vitamin D metabolism in all likelihood begins with 25(OH)D. As a result, the nutritional vitamin D status of the human fetus/neonate is totally dependent on the vitamin D stores of the mother [11]; thus, if the mother is hypovitaminotic D, her fetus will experience depleted vitamin D exposure throughout gestation [14]. 

While the demands of the fetus on the maternal system for calcium increase throughout pregnancy, the demands for vitamin D do not appear to change. The main determinants of calcium homeostasis during pregnancy are parathyroid hormone, calcitonin, and the active form of vitamin D—1,25(OH)_2_D (whose synthesis is preferentially maintained even during times of vitamin D deficiency by upregulation of enzymes for improved utilization of whatever 25(OH)D is available). The principal maternal adjustment to the greater calcium requirements during pregnancy appears to be increasing PTH, which maintains the serum calcium concentration in the face of a falling albumin level, an expanding extracellular fluid volume, and increasing renal excretion and placental calcium transfer [15, 16]. The placenta itself is responsible for active transport of calcium ions, making the fetus hypercalcemic relative to the mother, thereby stimulating fetal calcitonin and suppressing fetal PTH secretion [16]. 

With respect to vitamin D, the placenta and/or deciduas are active extrarenal sites of conversion of 25(OH)D as they contain 1*α*-hydroxylase, providing a source of 1,25(OH)_2_D for the fetus. Despite an expanding intravascular volume during pregnancy, maternal vitamin D status shows seasonal variation but beyond the typical factors that influence vitamin D status in adults (such as sunlight exposure and BMI), vitamin D status does not fluctuate during pregnancy and once supplemented, maternal blood levels reach steady-state within 2 months [15]. These aspects of calcium and vitamin D metabolism are important in the understanding of vitamin D sufficiency during pregnancy and the premise that the requirements of vitamin D are stable throughout gestation.

Existing guidelines recommend that pregnant women receive 400 IU of vitamin D per day via an oral supplement [17, 18]. Among African-American women who receive supplementation of this amount, however, the rate of hypovitaminosis D (defined conservatively as the circulating 25-hydroxy-vitamin D [25(OH)D] level <15 ng/mL or <37.5 nmol/L) is still 28% [19]. The incidence of rickets, a pathology which can develop among children with hypovitaminosis D, increased during the past two decades, especially among populations with higher levels of skin pigmentation [20–22]. While rickets has been traditionally thought to be the main sequelae of vitamin D deficiency, numerous other long-latency disease states (such as multiple sclerosis [23–25], rheumatoid arthritis [26–29], type 1 and 2 diabetes [30–34], cardiovascular disease [35, 36], various cancers [37–43], and systemic lupus erythematosus [44], to name but a few) as well as acute infections such as upper respiratory illnesses [45] and tuberculosis [46, 47] are now linked with vitamin D deficiency that are explained by alterations in immune function [47–49]. Those specific to the pregnant woman and her fetus include impaired fetal growth [50–53], altered development of dentition manifested in early childhood, hypocalcemia—and rarely—neonatal seizures [54–56], and maternal preeclampsia [57, 58]. Studies are currently underway to determine the optimum supplementation dose of vitamin D among pregnant women (clinicaltrials.gov: NCT00412087 and NCT00292591). Once the optimum dose is determined, the treatment effect of this intervention will be modified by the level of nonadherence among the patient population. The clinical implications of nonadherence are numerous and well recognized [59–61].

Adherence to study medication is often measured by a calculation of pill count. This is an inexpensive measure of compliance, but the data may be unreliable and often missing. For example, in a 2005 study of prenatal supplements 56% of subjects remembered to return only one monthly pill bottle during the duration of the 2-3 month study [62]. With this method, researchers are not only depending on patients to return bottles, but not to alter the number of remaining doses as well. In a 2001 study of protease inhibitor regimen adherence among HIV patients, pill count measures estimated adherence at 83%, but electronic monitors on the pill bottles revealed that true adherence was only 63% [63]. 

Adherence affects outcomes in all treatments and among all groups, having an impact on public health. The purposes of this study were to define parameters for measuring adherence to vitamin D supplementation by measures of serum metabolite, which could then be applied to other medication regimens, and to examine the association between adherence as determined by serum metabolites and adherence as determined by patients' pill counts.

## 2. Methods

### 2.1. Study Design

Data were obtained from a large NIH-supported (#R01 HD043921) randomized, double-blind, placebo-control trial of vitamin D supplements in pregnant women that took place at the Medical University of South Carolina (MUSC). This paper, however, describes an observational analysis of a subset of subjects in the original trial. This research protocol was approved by the MUSC Institutional Review Board for Human Research (HR#10727). 

Women entered the study at or before 14 weeks gestation and continued their supplementation throughout pregnancy. The women participants had been stratified by race and randomized into three groups, each of which received a 400 IU (control), 2000 IU, or 4000 IU dose of vitamin D_3_ once daily. Only the women in the 2000 IU and 4000 IU dose groups were included in these analyses, since only these doses were expected to have significant influence on subjects' serum 20(OH)D levels.

Subjects (self-identified as Caucasian, African-American, Hispanic/Latina, Asian, or American Indian) who carried singleton pregnancies were eligible for participation in the study. All subjects were patients at obstetric clinics at MUSC and participated in a monthly study visit as an extension of their regular prenatal checkup until delivery. They were also asked to return to the clinic three times for study visits after the birth of their infants. At each study visit, as a marker of vitamin D status, circulating levels of 25(OH)D and vitamin D_3_ were measured in patient serum. In addition, subjects were asked to bring their supplement bottle with them to their monthly visits, containing all unused supplements. 

### 2.2. Measures

The key variables of interest in this study were measured as (1) mean percent adherence by pill count and (2) adherence by serum metabolite levels. These values were derived for each subject based on data obtained across multiple time points.

#### 2.2.1. Mean Percent Adherence by Pill Count

Subjects were asked to bring their supplement containers, containing all unused doses, with them to each monthly study visit. The percentage adherence at each time point for each subject was determined by the following formula: 

(no. of pills dispensed – no. of pills returned)/(no. of elapsed days between dispense date and return date).

Using the formula above, some subjects' adherence levels were evaluated to be more than 100%. This could occur if women returned the pill bottle containing fewer doses than were expected in the given time interval. In such instances, we assigned each of these subjects a 100% adherence for the corresponding time interval. The mean percent adherence by pill count for each subject was then calculated by taking the mean of percent adherence for all visits for which pill count data were available for that subject. 

#### 2.2.2. Adherence by Serum Metabolite Levels

Defining adherence by serum metabolite levels was not a straightforward process. While there is a model in existence which calculates the predicted change in serum 25(OH)D for different doses of vitamin D supplement, this model was developed using data from men [64], and its applicability to a population of pregnant females is debatable. Therefore, we were faced with the challenge of establishing novel criteria for vitamin D supplement adherence by serum 25(OH)D levels; how we arrived at this definition is described below. 

The 25(OH)D level for each patient had been obtained at each monthly visit. In developing a definition of adherence by serum 25(OH)D levels, several assumptions were made a priori about what types of patterns of 25(OH)D levels would be exhibited by adherent and nonadherent patients based on current knowledge about the metabolism of vitamin D.

**Assumption 1**

Based on known pharmacokinetics of vitamin D [64], adherent patients' 25(OH)D levels would be expected to rise over time, eventually reaching a steady state after 2 months of therapy; nonadherent patients' 25(OH)D levels would be expected to be erratic and to exhibit variable patterns that would differ significantly from adherent patients' 25(OH)D levels over the same dosing interval. Therefore, the average of each subject's steady-state 25(OH)D levels (i.e., levels obtained at least 2 months after initiation of vitamin D therapy) was calculated, and the subject's baseline 25(OH)D level was subtracted from that value to obtain a value for the change in 25(OH)D level during the study (Change 25OHD).

**Assumption 2**

Change 25(OH)D would be greater among adherent patients when compared to nonadherent patients.

**Assumption 3**

Individual subjects who were adherent would tend to exhibit less variability in their 25(OH)D measures over time (i.e., our “steady-state” assumption). In other words, we assumed that relative fluctuations in adherent subjects' 25(OH)D levels would be lower than those of nonadherent subjects. The coefficient of variation (CV), defined as the standard deviation of a set of measurements divided by the arithmetic mean of the measurements [65, 66], was selected as a tool for use in comparing subjects with respect to the degree of variation observed in their serum 25(OH)D levels. Because the CV reflects the level of variation of each subject's measures relative to her mean, it provides, in effect, a way of standardizing the degree of variability across subjects with different means and standard deviations. A lower CV represents less relative variability and indicates consistency in level of supplementation received.

To summarize the previous assumptions, an adherent subject's level of serum 25(OH)D was expected not only to increase over the study period, but also to be fairly consistent after 2 months (time to achieve steady state).

Finally, a definition of adherence by serum metabolite levels was proposed based upon the above assumptions. A subject who met the following criteria was classified as adherent by her serum metabolite levels.

1. high increase from baseline: the subject's overall change in 25(OH)D level over the course of the study (baseline value to steady state concentration) was at or above the median among subjects taking that supplement dosage.
2. low relative variability: the subject's CV in her 25(OH)D level was less than or equal to the median across all study subjects.

*Other Variables*. Assigned dose was categorized by treatment group. Maternal race (classified as African American, Hispanic, and Caucasian), marital status, and highest education level were determined by self-identification on a questionnaire administered at enrollment. Maternal age was calculated as self-reported date of birth subtracted from date of enrollment. Season at enrollment was determined by season (Winter, Spring, Summer, Fall) at first visit date. The median value was determined, and a dichotomous variable was created defining women as darker or lighter than the median subject skin pigmentation.

### 2.3. Statistical Analyses

The sample of subjects included in our analyses provided approximately 80% power to detect an odds ratio as small as 1.55, assuming 2-sided hypothesis testing and an alpha level of 0.05. Since the only way to assess whether mothers achieved steady state during the study was to examine mothers with repeated pill counts and serum measurements, an inclusion requisite established a priori for the study was inclusion of mothers who were seen monthly throughout their pregnancy. Thus, for the subjects included in our study, response rates were all approximately 100%.

To create the model, descriptive statistics pertaining to the adherence measures were first calculated for all patient characteristic subgroups. Next, a logistic regression model was created to examine whether mean percent adherence by pill count was significantly associated with adherence by serum metabolite levels, while controlling for age, race, season at enrollment, and baseline body mass index (BMI). Finally, a sensitivity analysis was performed, in which the limits on serum 25(OH)D change from baseline (i.e., >median) and coefficient of variation (i.e., <median) were varied to examine whether our findings were sensitive to the cutoffs selected for our novel definition of adherence by serum metabolite levels. The additional definitions of adherence incorporated cutoffs of the 25th and 75th percentile for serum 25(OH)D change from baseline (as opposed to using the median, or 50th percentile), along with the 75th percentile for the coefficient of variation. For these sensitivity analyses, additional multivariate logistic regression models were created for each combination of cutoffs for change and coefficient of variation, treating the revised novel definition of adherence as the dependent variable of interest. 

## 3. Results

A total of 161 subjects with available data were included in the study. Descriptive statistics for each subgroup of subjects is presented in Table 1. The mean (SD) baseline serum 25(OH)D level was 24.4 (8.4) ng/mL across all subjects, increasing, on average, by 19.0 (11.4) ng/mL to their steady-state value, with higher changes observed in the higher dose group, as expected. By pill count, subjects were adherent 82.9% of the time, while only 46.6% were adherent using our novel definition based on serum 25(OH)D levels.

Table 2 summarizes the results of the logistic regression model associated with our primary analysis. The analysis revealed that mean percentage of adherence by pill count was not significantly associated with adherence as measured by serum metabolite levels (odds ratio [OR] = 1.2, 95% confidence interval [CI] = 0.9 to 1.6). Of the other subject characteristics included in the model, the only variable exhibiting a significant association with adherence by serum metabolite levels was the subject dose group, with the odds of adherence among patients in the 2000 IU dose group being about half that of patients in the 4000 IU dose group (OR = 0.5, 95% CI = 0.2 to 0.9). The generalized R-square value for the model was 10.5%, indicating that much of the variation in adherence (by serum 25(OH)D) was not explained by pill count or the other variables in the model. Sensitivity analyses (data not shown) revealed that our findings were not sensitive to the additional cutoff limits used within our novel definition of adherence by serum metabolite levels (i.e., using the 25th and 75th percentiles for the cutoff for serum 25(OH)D change from baseline, and using the 75th percentile for the cutoff for the coefficient of variation).

## 4. Discussion

The importance of adherence to medication has been recognized in clinical trials and daily clinical practice alike. In order to properly evaluate the effects of medications, it is vital to determine whether they are taken as prescribed. The most frequent manner to evaluate adherence has been pill count, which offers advantages such as low cost and simplicity of collection and calculation, but yet has the disadvantages of frequently missing data and possible manipulation by subjects [67–73]. In this trial, we sought to determine whether there was a significant association between adherence to vitamin D supplementation as measured by pill count and assessment of a novel measure of adherence based upon serum levels of vitamin D metabolites in a pregnant population.

The results of the present study offer a new perspective on the determination of efficacy in clinical research. Currently, the efficacy of medications and supplements is determined primarily via randomized controlled clinical trials, in which subject adherence is assumed or measured via pill count and results are analyzed on an intent-to-treat basis as a measure of a medication's effectiveness. If there were an objective laboratory value by which adherence could more accurately be determined, studies could include the most adherent patients in the analysis to get a clearer picture of efficacy without the dilution of the effect by nonadherent patients.

According to our findings, while there was a persistently positive odds ratio between the pill count measure of adherence and our novel definition of adherence by serum metabolite levels, this association was not significant for any of the proposed definitions (Table 2). This confirms previous studies which showed that pill count data are prone to errors [67, 69, 73]. Our analytic technique, however, is unique in that it utilizes the pharmacokinetics of vitamin D metabolism to predict adherence, and the data are then compared to pill counts. Because maternal circulating 25(OH)D is not affected by gestational age per se, the stability of vitamin D during pregnancy allowed for analysis of adherence once steady state was achieved throughout gestation [15, 16]. Other markers such as 1,25(OH)_2_D, PTH, or calcitonin would not offer such stability as they change throughout gestation [16].

Strengths of this study include the variety of both pill count-based and serum metabolite-based definitions of compliance. The study is strengthened by the consistency of findings across multiple definitions on sensitivity analysis. In addition, the subject population in this study was composed exclusively of pregnant women, which are generally more adherent to treatment regimen than is the general population [74, 75]. 

A limitation of the study was missing pill count data, which limited the sample size. In fact, 10% of the original subject population was eliminated from this analysis at the outset because the subjects did not have a single time point at which pill count data was available. The majority of patients did not have pill count data for all time points, meaning that their general level of adherence was based on time points at which data were available. This may not be accurate, as an interim lack of adherence could have contributed to not returning the pill bottle at certain time points. There could also be unknown other factors, which could both affect adherence and the likelihood of remembering to bring the pill bottle to the next appointment. The lack of data is a persistent problem in pill count adherence analyses, and one which could be alleviated in the future by changing the way adherence is measured in clinical trials.

An unexplained finding in this study was the significant difference with adherence by subject dose group, with the odds of adherence among patients in the 2000 IU dose group being about half that of patients in the 4000 IU dose group. This may be a spurious finding that would disappear with further analysis of a larger sample, or it may be an artifact of our definition that we cannot otherwise explain. To resolve this issue, this finding needs to be examined more thoroughly in a larger sample of subjects.

In summary, based upon a novel definition of adherence that incorporates serial measurements of serum metabolite levels, we found that pill count was not a reliable predictor of adherence to protocol. These findings call into question the way that adherence is typically measured in clinical research, which also has implications in the determination of efficacy of medications or supplements under study. This study also offers an alternative approach to measuring compliance for supplements and medications with long half-lives. This approach could potentially be used clinically to monitor patient adherence to prescribed regimens of such supplements or medications, much as HbA1C levels are currently used to monitor long-term blood glucose control in diabetic patients.

## Figures and Tables

**Table 1:** Subject characteristics by serum 25(OH)D and adherence measurements.

Subject characteristic	*N*	Baseline serum 25(OH)D, ng/mL, mean (SD)	Change in serum 25(OH)D, ng/mL, mean (SD)	CV for steady-state serum 25(OH)D measurements, mean (SD)	Mean percent adherent by pill count data, mean (SD)	Percent adherent by the novel serum 25(OH)D definition
Maternal age						
<20	6	18.5 (10.3)	+22.4 (17.4)	18.9% (9.0%)	74.3% (16.6%)	33.3%
20–25	50	23.2 (7.9)	+23.2 (7.9)	17.5% (8.4%)	81.4% (15.3%)	46.0%
26–30	44	23.5 (8.7)	+23.5 (8.7)	18.4% (8.2%)	83.4% (12.8%)	47.7%
≥30	61	26.6 (7.9)	+26.6 (7.9)	17.9% (10.1%)	84.7% (12.6%)	47.5%
Race						
Black	32	17.3 (7.1)	+21.1 (13.3)	20.4% (11.6%)	74.8% (13.4%)	53.1%
Latina	76	24.5 (7.6)	+18.1 (11.0)	16.7% (8.3%)	86.1% (13.4%)	43.4%
White	53	28.6 (7.2)	+18.9 (10.6)	18.2% (7.8%)	83.2% (12.7%)	47.2%
Body mass index						
<20	15	29.5 (7.9)	+17.5 (9.6)	16.8% (8.5%)	90.0% (6.1%)	33.3%
20–25	56	25.4 (9.3)	+18.0 (12.5)	17.7% (9.8%)	82.6% (11.5%)	44.6%
26–30	39	23.0 (7.8)	+22.4 (11.9)	17.2% (7.6%)	86.0% (10.5%)	53.8%
≥30	40	23.3 (7.5)	+18.2 (9.8)	19.2% (8.1%)	80.3% (16.0%)	47.5%
Season at enrollment						
Winter	34	24.3 (8.4)	+20.2 (12.2)	16.9% (9.4%)	83.4% (12.6%)	50.0%
Spring	53	22.6 (7.8)	+20.1 (10.9)	18.8% (10.7%)	83.7% (15.9%)	43.4%
Summer	40	25.2 (7.3)	+17.2 (11.2)	17.7% (7.3%)	84.5% (12.3%)	40.0%
Fall	34	26.5 (9.9)	+18.2 (11.5)	17.9% (7.4%)	79.4% (12.7%)	55.9%
Dose						
2000 IU	85	24.5 (8.7)	+15.5 (10.7)	18.5% (8.9%)	82.3% (13.3%)	37.6%
4000 IU	76	24.4 (8.0)	+22.9 (10.8)	17.2% (9.1%)	83.6% (14.3%)	56.6%
Adherence by pill count						
<70%	25	25.7 (9.8)	+13.8 (10.1)	17.4% (9.0%)	57.8% (12.0%)	40.0%
>70%	136	24.2 (8.1)	+20.0 (11.4)	18.0% (9.0%)	87.5% (7.8%)	47.8%
All subjects	161	24.4 (8.4)	+19.0 (11.4)	17.9% (9.0%)	82.9% (13.7%)	46.6%

**Table 2:** Results of the primary logistic regression model predicting adherence as determined by the novel serum 25(OH)D definition.

Subject characteristic	*N*	Odd ratio	95% confidence interval	*P*-value
Mean percent adherence (as measured by pill count)	161	1.2*	(0.9, 1.6)	.29
Maternal age	161	1.0^†^	(1.0, 1.1)	.45
Race				
Black	32	1.8	(0.6, 5.0)	.20
Latina	76	1.0	(0.4, 2.3)	.41
White	53	Referent	—	
Body mass index	161	1.0^‡^	(1.0, 1.1)	.22
Dose				
2000 IU	85	0.5	(0.2, 0.9)	.02
4000 IU	76	Referent	—	
Season at enrollment				
Spring	53	0.7	(0.3, 1.8)	.30
Summer	40	0.7	(0.2, 1.8)	.24
Fall	34	1.7	(0.6, 4.9)	.08
Winter	34	Referent	—	

*Mean percent adherence was treated as a continuous measure in the model; however, this variable was coded such that the odds ratio reflects the increase in odds associated with a 10-percent increase in mean percent adherence.

^†^Maternal age at baseline was treated as a continuous measure in the model; however, this variable was coded such that the odds ratio reflects the increase in odds associated with a 1-year increase in age.

^‡^Body mass index was treated as a continuous measure in the model; however, this variable was coded such that the odds ratio reflects the increase in odds associated with a 1-unit increase in body mass index.

## References

[1] Vieth R, Bischoff-Ferrari H, Boucher BJ (2007). The urgent need to recommend an intake of vitamin D that is effective. *American Journal of Clinical Nutrition*.

[2] Holick MF (2007). Medical progress: vitamin D deficiency. *The New England Journal of Medicine*.

[3] Matsuoka LY, Wortsman J, Haddad JG, Hollis BW (1989). In vivo threshold for cutaneous synthesis of vitamin D_3_. *Journal of Laboratory and Clinical Medicine*.

[4] Matsuoka LY, Wortsman J, Hollis BW (1990). Suntanning and cutaneous synthesis of vitamin D_3_. *Journal of Laboratory and Clinical Medicine*.

[5] Matsuoka LY, Wortsman J, Hollis BW (1990). Use of topical sunscreen for the evaluation of regional synthesis of vitamin D_3_. *Journal of the American Academy of Dermatology*.

[6] Matsuoka LY, Wortsman J, Haddad JG, Hollis BW (1990). Skin types and epidermal photosynthesis of vitamin D_3_. *Journal of the American Academy of Dermatology*.

[7] Matsuoka LY, Wortsman J, Haddad JG, Kolm P, Hollis BW (1991). Racial pigmentation and the cutaneous synthesis of vitamin D. *Archives of Dermatology*.

[8] Bouillon R, Van Baelen H, DeMoor D (1977). 25-hydroxy-vitamin D and its binding protein in maternal and cord serum. *The Journal of Clinical Endocrinology & Metabolism*.

[9] Bouillon R, van Assche FA, van Baelen H, Heyns W, De Moor P (1981). Influence of the vitamin D-binding protein on the serum concentration of 1,25-dihydroxyvitamin D_3_. Significance of the free 1,25-dihydroxyvitamin D3 concentration. *The Journal of Clinical Investigation*.

[10] Markestad T, Aksnes L, Ulstein M, Aarskog D (1984). 25-hydroxyvitamin D and 1,25-dihydroxyvitamin D of D_2_ and D_3_ origin in maternal and umbilical cord serum after vitamin D_2_ supplementation in human pregnancy. *American Journal of Clinical Nutrition*.

[11] Hollis BW, Pittard WB (1984). Evaluation of the total fetomaternal vitamin D relationships at term: evidence for racial differences. *The Journal of Clinical Endocrinology & Metabolism*.

[12] Greer FR, Hollis BW, Napoli JL (1984). High concentrations of vitamin D_2_ in human milk associated with pharmacologic doses of vitamin D_2_. *Journal of Pediatrics*.

[13] Goff JP, Horst RL, Littledike ET (1984). Effect of sow vitamin D status at parturition on the vitamin D status of neonatal piglets. *Journal of Nutrition*.

[14] Hollis BW, Wagner CL (2004). Assessment of dietary vitamin D requirements during pregnancy and lactation. *American Journal of Clinical Nutrition*.

[15] Ardawi MSM, Nasrat HAN, BA’Aqueel HS (1997). Calcium-regulating hormones and parathyroid hormone-related peptide in normal human pregnancy and postpartum: a longitudinal study. *European Journal of Endocrinology*.

[16] Pitkin RM (1985). Calcium metabolism in pregnancy and the perinatal period: a review. *American Journal of Obstetrics and Gynecology*.

[17] Institute of Medicine (1990). *Calcium, Vitamin D, and Magnesium. Subcommittee on Nutritional Status and Weight Gain during Pregnancy: Nutrition During Pregnancy*.

[18] Institute of Medicine (1991). *Nutrition During Lactation. Subcommittee on Nutrition During Lactation Food and Nutrition Board*.

[19] Nesby-O’Dell S, Scanlon KS, Cogswell ME (2002). Hypovitaminosis D prevalence and determinants among African American and white women of reproductive age: third National Health and Nutrition Examination Survey, 1988–1994. *American Journal of Clinical Nutrition*.

[20] Kreiter S (2001). The reemergence of vitamin D deficiency rickets-the need for vitamin D supplementation. *AMB News and Views: The Newsletter of the Academy of Breastfeeding Medicine*.

[21] Gartner L, Greer F (2003). American Academy of Pediatrics. Section on breastfeeding medicine and committee on nutrition. Prevention of rickets and vitamin D deficiency: new guidelines for vitamin D intake. *Pediatrics*.

[22] Wagner CL, Greer FR (2008). Prevention of rickets and vitamin D deficiency in infants, children, and adolescents. *Pediatrics*.

[23] Cantorna MT, Hayes CE, Deluca HF (1996). 1,25-dihydroxyvitamin D3 reversibly blocks the progression of relapsing encephalomyelitis, a model of multiple sclerosis. *Proceedings of the National Academy of Sciences of the United States of America*.

[24] Munger KL, Levin LI, Hollis BW, Howard NS, Ascherio A (2006). Serum 25-hydroxyvitamin D levels and risk of multiple sclerosis. *Journal of the American Medical Association*.

[25] Correale J, Ysrraelit MC, Gaitn MI (2009). Immunomodulatory effects of Vitamin D in multiple sclerosis. *Brain*.

[26] Merlino LA, Curtis J, Mikuls TR, Cerhan JR, Criswell LA, Saag KG (2004). Vitamin D intake is inversely associated with rheumatoid arthritis: results from the Iowa women’s health study. *Arthritis and Rheumatism*.

[27] Costenbader KH, Feskanich D, Holmes M, Karlson EW, Benito-Garcia E (2008). Vitamin D intake and risks of systemic lupus erythematosus and rheumatoid arthritis in women. *Annals of the Rheumatic Diseases*.

[28] Cutolo M, Otsa K, Yprus M, Seriolo B (2007). Vitamin D and rheumatoid arthritis: comment on the letter by Nielen et al. *Arthritis and Rheumatism*.

[29] Muller K, Kriegbaum NJ, Baslund B, Sorensen OH, Thymann M, Bentzen K (1995). Vitamin D3 metabolism in patients with rheumatic diseases: low serum levels of 25-hydroxyvitamin D3 in patients with systemic lupus erythematosus. *Clinical Rheumatology*.

[30] Dahlquist G (1999). Vitamin D supplement in early childhood and risk for type I (insulin-dependent) diabetes mellitus. *Diabetologia*.

[31] Stene LC, Ulriksen J, Magnus P, Joner G (2000). Use of cod liver oil during pregnancy associated with lower risk of type I diabetes in the offspring. *Diabetologia*.

[32] Hypponen E, Laara E, Reunanen A, Jarvelin M-R, Virtanen SM (2001). Intake of vitamin D and risk of type 1 diabetes: a birth-cohort study. *The Lancet*.

[33] Pittas AG, Dawson-Hughes B, Li T (2006). Vitamin D and calcium intake in relation to type 2 diabetes in women. *Diabetes Care*.

[34] Tangpricha V, Pearce EN, Chen TC, Holick MF (2002). Vitamin D insufficiency among free-living healthy young adults. *American Journal of Medicine*.

[35] Wang TJ, Pencina MJ, Booth SL (2008). Vitamin D deficiency and risk of cardiovascular disease. *Circulation*.

[36] Giovannucci E, Liu Y, Hollis BW, Rimm EB (2008). 25-hydroxyvitamin D and risk of myocardial infarction in men: a prospective study. *Archives of Internal Medicine*.

[37] Rohan T (2007). Epidemiological studies of vitamin D and breast cancer. *Nutrition Reviews*.

[38] Tworoger SS, Lee I-M, Buring JE, Rosner B, Hollis BW, Hankinson SE (2007). Plasma 25-hydroxyvitamin D and 1,25-dihydroxyvitamin D and risk of incident ovarian cancer. *Cancer Epidemiology Biomarkers and Prevention*.

[39] Weigel NL (2007). Interactions between vitamin D and androgen receptor signaling in prostate cancer cells. *Nutrition Reviews*.

[40] Wu K, Feskanich D, Fuchs CS, Willett WC, Hollis BW, Giovannucci EL (2007). A nested case-control study of plasma 25-hydroxyvitamin D concentrations and risk of colorectal cancer. *Journal of the National Cancer Institute*.

[41] Zhou W, Heist RS, Liu G (2007). Circulating 25-hydroxyvitamin D levels predict survival in early-stage non-small-cell lung cancer patients. *Journal of Clinical Oncology*.

[42] Garland CF, Garland FC, Shaw EK, Comstock GW, Helsing KJ, Gorham ED (1989). Serum 25-hydroxyvitamin D and colon cancer: eight-year prospective study. *The Lancet*.

[43] Lefkowitz ES, Garland CF (1994). Sunlight, vitamin D, and ovarian cancer mortality rates in US women. *International Journal of Epidemiology*.

[44] Kamen D, Aranow C (2008). Vitamin D in systemic lupus erythematosus. *Current Opinion in Rheumatology*.

[45] Laaksi I, Ruohola J-P, Tuohimaa P (2007). An association of serum vitamin D concentrations <40 nmol/L with acute respiratory tract infection in young Finnish men. *American Journal of Clinical Nutrition*.

[46] Martineau AR, Wilkinson RJ, Wilkinson KA (2007). A single dose of vitamin D enhances immunity to mycobacteria. *American Journal of Respiratory and Critical Care Medicine*.

[47] Liu PT, Stenger S, Tang DH, Modlin RL (2007). Cutting edge: vitamin D-mediated human antimicrobial activity against *Mycobacterium tuberculosis* is dependent on the induction of cathelicidin. *Journal of Immunology*.

[48] Liu PT, Stenger S, Li H (2006). Toll-like receptor triggering of a vitamin D-mediated human antimicrobial response. *Science*.

[49] Enioutina EY, Bareyan D, Daynes RA (2009). TLR-induced local metabolism of vitamin D3 plays an important role in the diversification of adaptive immune responses. *Journal of Immunology*.

[50] Delvin EE, Salle BL, Glorieux FH, Adeleine P, David LS (1986). Vitamin D supplementation during pregnancy: effect on neonatal calcium homeostasis. *Journal of Pediatrics*.

[51] Brooke OG, Brown IRF, Bone CDM (1980). Vitamin D supplements in pregnant Asian women: effects on calcium status and fetal growth. *British Medical Journal*.

[52] Brooke OG, Butters F, Wood C (1981). Intrauterine vitamin D nutrition and postnatal growth in Asian infants. *British Medical Journal*.

[53] Maxwell JD, Ang L, Brooke OG, Brown IRF (1981). Vitamin D supplements enhance weight gain and nutritional status in pregnant Asians. *British Journal of Obstetrics and Gynaecology*.

[54] Rosen JF, Roginsky M, Nathenson G, Finberg L (1974). 25-hydroxyvitamin D. Plasma levels in mothers and their premature infants with neonatal hypocalcemia. *American Journal of Diseases of Children*.

[55] Gessner BD, deSchweinitz E, Petersen KM, Lewandowski C (1997). Nutritional rickets among breast-fed black and Alaska Native children. *Alaska Medicine*.

[56] Hatun S, Ozkan B, Orbak Z (2005). Vitamin D deficiency in early infancy. *Journal of Nutrition*.

[57] Hypponen E (2005). Vitamin D for the prevention of preeclampsia? A hypothesis. *Nutrition Reviews*.

[58] Bodnar LM, Catov JM, Simhan HN, Holick MF, Powers RW, Roberts JM (2007). Maternal vitamin D deficiency increases the risk of preeclampsia. *The Journal of Clinical Endocrinology & Metabolism*.

[59] Osterberg L, Blaschke T (2005). Adherence to medication. *The New England Journal of Medicine*.

[60] Baker H, Napthine R (1994). The compliance conundrum. *Australian Nursing Journal*.

[61] Cramer JA (1995). Relationship between medication compliance and medical outcomes. *American Journal of Health-System Pharmacy*.

[62] Jasti S, Siega-Riz AM, Cogswell ME, Hartzema AG, Bentley ME (2005). Pill count adherence to prenatal multivitamin/mineral supplement use among low-income women. *Journal of Nutrition*.

[63] Liu H, Golin CE, Miller LG (2001). A comparison study of multiple measures of adherence to HIV protease inhibitors. *Annals of Internal Medicine*.

[64] Heaney RP, Davies KM, Chen TC, Holick MF, Barger-Lux MJ (2003). Human serum 25-hydroxycholecalciferol response to extended oral dosing with cholecalciferol. *American Journal of Clinical Nutrition*.

[65] Rosner B, Spiegelman D, Willett WC (1990). Correction of logistic regression relative risk estimates and confidence intervals for measurement error: the case of multiple covariates measured with error. *American Journal of Epidemiology*.

[66] Rosner B (2007). *Fundamentals of Biostatistics*.

[67] Farmer KC (1999). Methods for measuring and monitoring medication regimen adherence in clinical trials and clinical practice. *Clinical Therapeutics*.

[68] Chao D, Foy CG, Farmer D (2000). Exercise adherence among older adults: challenges and strategies. *Controlled Clinical Trials*.

[69] Mihalko SL, Brenes GA, Farmer DF, Katula JA, Balkrishnan R, Bowen DJ (2004). Challenges and innovations in enhancing adherence. *Controlled Clinical Trials*.

[70] Rathbun RC, Farmer KC, Stephens JR, Lockhart SM (2005). Impact of an adherence clinic on behavioral outcomes and virologic response in treatment of HIV infection: a prospective, randomized, controlled pilot study. *Clinical Therapeutics*.

[71] Mukherjee JS, Ivers L, Leandre F, Farmer P, Behforouz H (2006). Antiretroviral therapy in resource-poor settings: decreasing barriers to access and promoting adherence. *Journal of Acquired Immune Deficiency Syndromes*.

[72] Rathbun RC, Farmer KC, Lockhart SM, Stephens JR (2007). Validity of a stage of change instrument in assessing medication adherence in indigent patients with HIV infection. *Annals of Pharmacotherapy*.

[73] Farmer AJ, Prevost AT, Hardeman W (2008). Protocol for SAMS (Support and Advice for Medication Study): a randomised controlled trial of an intervention to support patients with type 2 diabetes with adherence to medication. *BMC Family Practice*.

[74] Stevenson RE, Allen WP, Pai GS (2000). Decline in prevalence of neural tube defects in a high-risk region of the United States. *Pediatrics*.

[75] Bardeguez AD, Lindsey JC, Shannon M (2008). Adherence to antiretrovirals among US women during and after pregnancy. *Journal of Acquired Immune Deficiency Syndromes*.

